# A Mitochondrial Genome of Rhyparochromidae (Hemiptera: Heteroptera) and a Comparative Analysis of Related Mitochondrial Genomes

**DOI:** 10.1038/srep35175

**Published:** 2016-10-19

**Authors:** Teng Li, Jie Yang, Yinwan Li, Ying Cui, Qiang Xie, Wenjun Bu, David M. Hillis

**Affiliations:** 1 Institute of Zoology and Developmental Biology, College of Life Sciences, Lanzhou University, 222 Tianshui South Road, Lanzhou 730000, China; 2 Institute of Entomology, College of Life Sciences, Nankai University, 94 Weijin Road, Tianjin 300071, China; 3 Department of Integrative Biology, University of Texas at Austin, Austin, TX 78712, USA

## Abstract

The Rhyparochromidae, the largest family of Lygaeoidea, encompasses more than 1,850 described species, but no mitochondrial genome has been sequenced to date. Here we describe the first mitochondrial genome for Rhyparochromidae: a complete mitochondrial genome of *Panaorus albomaculatus* (Scott, 1874). This mitochondrial genome is comprised of 16,345 bp, and contains the expected 37 genes and control region. The majority of the control region is made up of a large tandem-repeat region, which has a novel pattern not previously observed in other insects. The tandem-repeats region of *P. albomaculatus* consists of 53 tandem duplications (including one partial repeat), which is the largest number of tandem repeats among all the known insect mitochondrial genomes. Slipped-strand mispairing during replication is likely to have generated this novel pattern of tandem repeats. Comparative analysis of tRNA gene families in sequenced Pentatomomorpha and Lygaeoidea species shows that the pattern of nucleotide conservation is markedly higher on the J-strand. Phylogenetic reconstruction based on mitochondrial genomes suggests that Rhyparochromidae is not the sister group to all the remaining Lygaeoidea, and supports the monophyly of Lygaeoidea.

The Rhyparochromidae, or dirt-colored seed bugs, are a relatively large group of Heteroptera and the largest family of Lygaeoidea (Hemiptera: Heteroptera)^1^. Rhyparochromidae includes two subfamilies (Plinthisinae and Rhyparochrominae), 372 genera, and more than 1,850 species^2^. The family was established by Amyot and Serville (1843) as Rhyparochromides, when it was regarded as a subfamily of the Lygaeidae^3^. Henry (1997) raised it to a family status and considered it as the basal group of Lygaeoidea based on morphological characters^1^. Dong and Zheng (1997), also using morphological characters, considered Rhyparochromidae as the sister group of Pyrrhocoroidea, which in turn forms the sister clade of the remaining Lygaeoidea^4^. In later molecular studies, analyses of 18S rDNA^5^, 18S + 28S rDNAs^6^, 16S rDNA^7^ and six Hox genes^8^ suggested that Rhyparochromidae is not the sister group to all the remaining Lygaeoidea. However, the phylogenetic relationships of Rhyparochromidae within Lygaeoidea have not been analyzed using mitochondrial genome (mt-genome) sequences to date, because no mt-genome data have been reported for the Rhyparochromidae.

Over the past decade, the mt-genome has become the most extensively used genomic resource in phylogenomic analyses of insects^9^, owing to its compact size (typically 15–18 kb), the availability of near-universal primers across insects, and the rapid development of sequencing technologies^10^,^11^,^12^. However, mt-genome representation at the family level is still limited, especially in most species-rich groups such as Heteroptera.

The size of true bug (Hemiptera: Heteroptera) mt-genomes range from 14,688 bp in *Kleidocerys resedae resedae* (Lygaeidae)^13^ to 17,544 bp in *Nesidiocoris tenuis* (Miridae)^14^. The main source of size variation is the variable number and length of tandem repeats in the control region^15^. Tandem repetition has been widely observed in the mitochondrial control region in animals^16^, and these duplications are thought to play a role in gene transcription or DNA methylation^17^,^18^. Moreover, tandem repeat units can be added or subtracted by slipped-strand mispairing during replication^19^, and this type of mutation happens at a rate at least 100,000 times higher compared to the rate of simple point mutations^20^. In true bugs, tandem repeats have been found in many groups, especially at the 3′-end of the control region (near the *tRNA-I* sequence), although the size and copy number of repeat units differ greatly among different species^21^,^22^,^23^. Length of the repeat unit can range from several base pairs to more than 200 bp^24^, and the copy number can range from at least 2 to 20^23^.

Here we describe the complete sequence of the mitochondrial genome of *Panaorus albomaculatus*, which represents the first sequenced mt-genome of the dirt-colored seed bugs. We evaluate compositional biases, codon usage, and nucleotide composition of this mt-genome. We then investigate the phylogenetic position of Rhyparochromidae within Heteroptera based on 13 protein-coding genes (PCGs) within the mt-genome. We compare the tRNA gene families (and their secondary structures) with other sequenced Pentatomomorpha and Lygaeoidea species. We also analyze two rRNA secondary structures across the sequenced Heteroptera and Lygaeoidea mt-genomes. Furthermore, we report novel patterns and large numbers of tandem repeats in the control region of the *Panaorus albomaculatus* mt-genome.

## Results and Discussion

### Genome organization and structure

**Figure 1:**
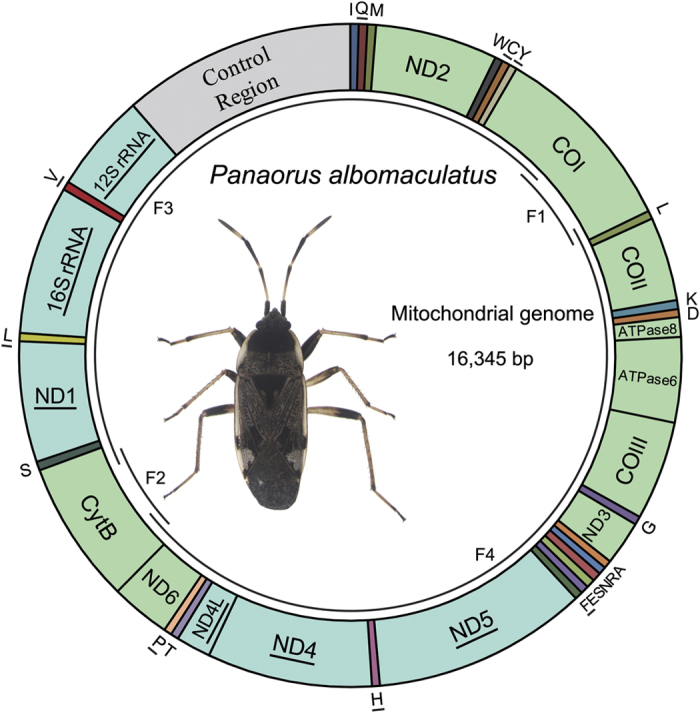
Structure of the mitochondrial genome of *Panaorus albomaculatus* (GenBank accession number KX216853). Gene names without an underline indicate genes transcribed from the majority strand (J-strand), and those with an underline indicate genes transcribed from the minority strand (N-strand). The tRNA genes are denoted by the color blocks and are named using single-letter amino acid abbreviations. Overlapping arcs (F1–F4) inside the circle indicate the PCR-amplified fragments.

The mitochondrial genome of *P. albomaculatus* contains 16,345 bp (GenBank Accession Number: KX216853; Fig. 1). The gene order of the 37 genes (including 13 PCGs, 22 tRNAs and two rRNAs) as well as the non-coding region (control region) is the same as observed in most other mt-genomes of true bugs^25^ (Table 1). The genome is relatively compact with gene overlaps at 16 gene junctions, involving a total of 75 bp. The longest overlap occurs between *tRNA-Ser* and ND1 and involves 20 bp. Two of the overlaps (between ATP8/ATP6 and ND4L/ND4) consist of the same seven nucleotides (ATGATAA). There are also 82 nucleotides in 4 intergenic spacers (in addition to the control region), which range in size from 1 to 77 bp.

### Protein coding genes

In the mt-genome of *P. albomaculatus*, eleven of the thirteen protein-coding genes initiate with ATN as the start codon (five start with ATT, four start with ATG, one starts with ATA, and one starts with ATC) (Table 1). The COI gene initiates with a non-traditional start codon, TTG, as previously observed in other true bugs^25^,^26^. The ND4L gene has the unusual start codon GTG, as previously reported in assassin bugs^27^ (Hemiptera: Reduviidae). Most protein coding genes end with a complete termination codon. Eight genes (ND2, COI, ATP8, ATP6, ND5, ND4L, ND6 and ND1) terminate with TAA, and two genes (ND3 and CytB) terminate with TAG. The remaining three PCGs (COII, COIII and ND4) are terminated with an incomplete stop codon T.

In Pentatomomorpha mt-genomes, most PCGs use the standard start codon (ATN), but we also observed other initiation codons of ATCA (ND2), CTG (ND1), GTG (COII, ND1, ND4L and ND6), and TTG (ATP8, COI, COII, ND1, ND4L, ND5 and ND6; see Supplementary Table S1). Many PCGs use multiple start codons, but three PCGs always initiate with only one start codon (COI with TTG, COIII and ND4 with ATG). Furthermore, several PCGs initiate with a start codon only found within Lygaeoidea mt-genomes (e.g., ATP6 start with ATT only in *Yemmalysus parallelus*). Most PCGs stop with termination codons TAA/TAG, but truncated termination codons T/TA are also used in Pentatomomorpha mt-genomes. The phenomenon of incomplete stop codons is common in insect mt-genomes and it is likely that these truncated stop codons are completed by posttranscriptional polyadenylation^28^.

We compared the rate of nonsynonymous substitutions (Ka), the rate of synonymous substitutions (Ks), and the ratio of Ka/Ks for each PCG among the 13 PCGs in Pentatomomorpha and Lygaeoidea mt-genomes (see Supplementary Figure S1). The results showed that the evolutionary patterns of 13 PCGs were highly consistent between Pentatomomorpha and Lygaeoidea, and shared many common features: (1) ATP8 has the highest evolutionary rate and can be used to analyze intraspecific relationships^29^, while COI appears to have the lowest evolutionary rate and has been used for DNA barcoding markers^30^. (2) The Ka/Ks ratios for all PCGs are less than 1, indicating that these genes are likely evolving primarily under the purifying selection^31^. (3) The uniformly low evolutionary rates and low Ka/Ks ratios (Ka/Ks < 0.2) for four genes (COI, COII, COIII and CytB) indicate strong purifying selection and evolutionary constraints in cytochrome c oxidase and cytochrome b^32^. (4) Negative correlations have been detected between the Ka/Ks ratios and the G + C content of each PCG in Pentatomomorpha (R^2^ = 0.918) and Lygaeoidea (R^2^ = 0. 891) mt-genomes (see Supplementary Figure S2), suggesting that the variation of G + C content probably causes the different evolutionary patterns among genes^22^. Additionally, most PCGs of Lygaeoidea mt-genomes had lower evolutionary rates than Pentatomomorpha.

### Nucleotide composition and codon usage

The nucleotide composition of the mt-genome of *P. albomaculatus* is significantly biased toward A/T. The overall A + T content of the J-strand is 76% (A = 44.4%, T = 31.6%, C = 14.5%, G = 9.5%; see Supplementary Table S2). This bias in A + T content falls within the known range for hemipteran mt-genomes (65.7% in *Bemisia afer* to 86.3% in *Aleurodicus dugesii*)^29^. The A + T content ranges from 75.1% for the PCGs, to 78.7% for the rRNAs. Among the PCGs, the lowest A + T content is 68.8% in COI, whereas the highest A + T content is 84% in ATP8. The nucleotide skew statistics^33^ for the mt-genome of *P. albomaculatus* reveal that the J-strand PCGs are AT-skewed and CG-skewed, whereas the N-strand PCGs are GC-skewed and TA-skewed. The observed strand bias is likely related to asymmetric mutation processes during replication^34^.

The overall A/T bias also influences codon usage. The A + T content of the third codon position (85.5%) is higher than either the first (71.3%) or second (68.2%) codon positions (see Supplementary Table S2). Moreover, the six most prevalent codons in *P. albomaculatus* (see Supplementary Table S3), Ile (ATT) (9.54%), Leu (TTA) (9.21%), Met (ATA) (7.87%), Phe (TTT) (7.41%), Asn (AAT) (4.92%), and Tyr (TAT) (4.48%) are all composed of A and/or T.

### Comparison of tRNAs in Pentatomomorpha and Lygaeoidea mt-genomes

**Figure 2:**
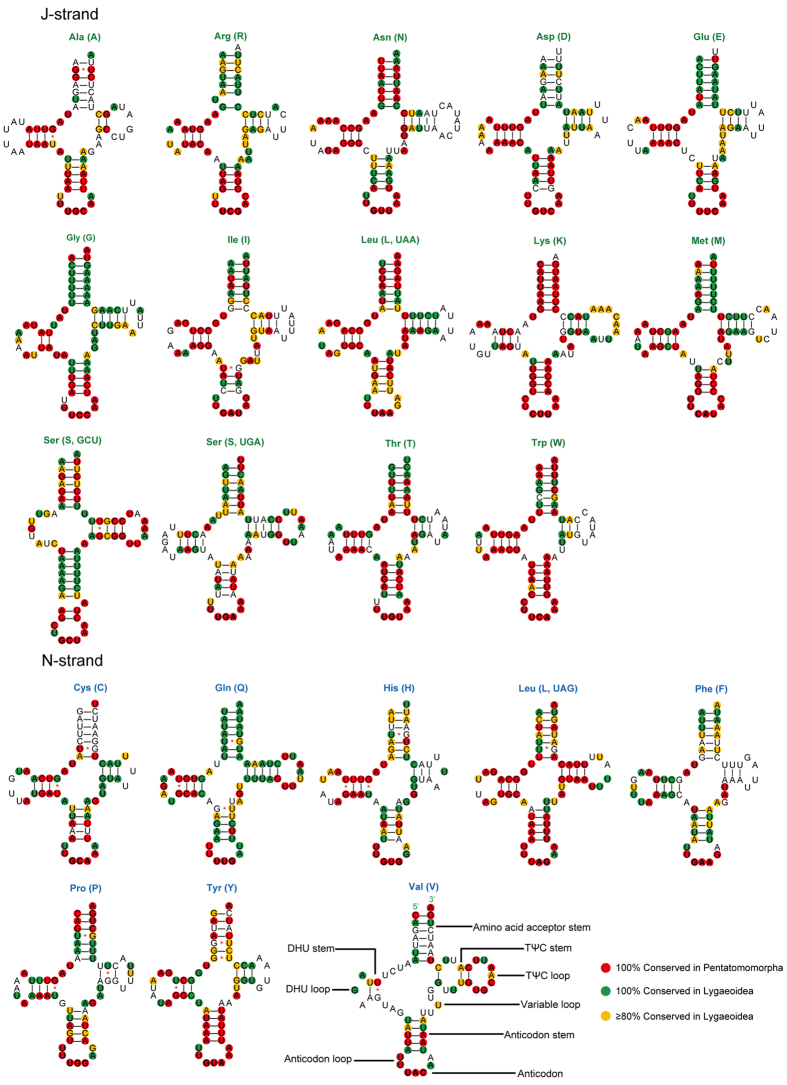
Putative secondary structure of the 22 tRNAs identified in the mitochondrial genome of *Panaorus albomaculatus*. The tRNAs are labeled with the abbreviations of their corresponding amino acids. Dashes indicate Watson-Crick base pairing and asterisks indicate G-U base pairing. Sites that are conserved 100% among sequenced Pentatomomorpha species are indicated with a red background. The 100% and > 80% conserved sites among sequenced Lygaeoidea species are indicated with green and orange backgrounds, respectively.

There are 22 tRNA genes in the mt-genome *P. albomaculatus* mt-genomes, as observed in other arthropod mt-geneomes. These genes range in length from 62 to 71 bp. Among these tRNAs, the *tRNA-Ser (GCU)* is the only one that does not fold into the typical cloverleaf secondary structure; the dihydrouridine (DHU) stem simply forms a loop (Fig. 2). Moreover, the predicted *tRNA-Ser (GCU)* anticodon stem is longer (9 bp vs. the normal 5 bp) and contains an unpaired nucleotide, as seen in many other insect mt-genomes^35^. A total of 20 unmatched G-U pairs exist in *P. albomaculatus* tRNA secondary structures. Of these, 16 mismatches are concentrated in 7 tRNAs that are encoded on the N-strand. These mismatches are common in arthropod mt-genomes; RNA editing processes are likely involved in correcting the mismatches^36^.

Most tRNA genes encoded on the J-strand are more conserved than those encoded on the N-strand, based on comparisons of aligned genes in Pentatomomorpha and Lygaeoidea (Fig. 2). Furthermore, some tRNAs (*tRNA-Gln*, *tRNA-Gly*, and *tRNA-Met*) within Lygaeoidea are much more conserved than they are within Pentatomomorpha, with changes mainly on the amino acid acceptor and TΨC stems. The anticodon stem and loop, and the DHU stem, are extremely conserved in most tRNAs within Pentatomomorpha, whereas most other tRNA regions have a high level of variation (the exceptions are the highly conserved TΨC stem of *tRNA-Leu* and acceptor stem of *tRNA-Lys*). The stems of each tRNA in Lygaeoidea mtDNA are more conserved than the remaining loops (TΨC, DHU and variable loops), except for several amino acid acceptor arms (*tRNA-Ala*, *tRNA-Asp*, *tRNA-Cys*, and *tRNA-Val*) and TΨC arms (*tRNA-His*, *tRNA-Phe* and *tRNA-Pro*). Most nucleotide substitutions are restricted to those loops (TΨC, DHU, and variable loops), which usually accompany insertion-deletion polymorphisms. The presence of indels at different taxonomic levels supports potential phylogenetic value of tRNA sequences in the study of insect phylogenetic relationships, especially when secondary structures are taken into account^21^,^37^.

**Figure 3:**
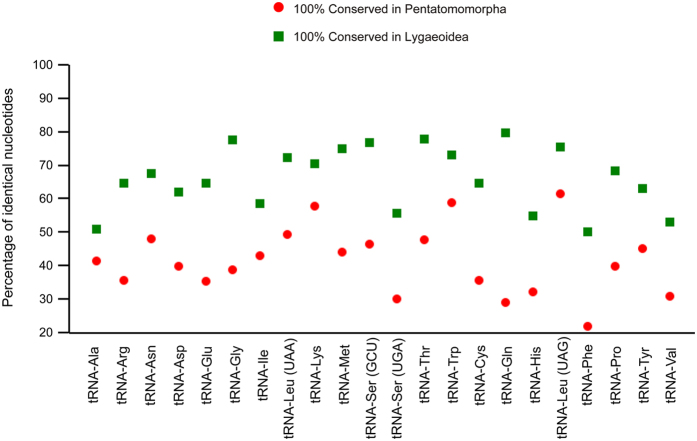
Conservation of tRNA families in Pentatomomorpha and Lygaeoidea mitochondrial genomes. The percentage of identical nucleotides for each tRNA family was inferred from a multiple alignment produced with CLUSTAL X^61^ and refined manually, taking into account the secondary structure.

We determined the percentage of identical nucleotides (INP%) for each tRNA family in Pentatomomorpha and Lygaeoidea mt-genomes, respectively (Fig. 3). The sequences and secondary structures of the *tRNA-Leu (TAG)*, *tRNA-Trp*, and *tRNA-Lys* genes are highly conserved among most Pentatomomorpha mt-genomes (INP% > 55). In contrast, the most conserved tRNAs in Lygaeoidea mt-genomes (INP% > 75) are *tRNA-Gln*, *tRNA-Thr*, *tRNA-Gly*, *tRNA-Ser (GCT)*, and *tRNA-Leu (TAG).* The highest level of nucleotide conservation in Lygaeoidea mt-genomes occurs in *tRNA-Gln* (INP% = 79.8), which is a much more variable gene in Pentatomomorpha (INP% = 28.9). Although the pattern of nucleotide conservation is markedly higher on the J-strand, the highest INP% values of *tRNA-Leu (TAG)* in Pentatomomorpha (INP% = 61.5) and *tRNA-Gln* (INP% = 79.8) in Lygaeoidea are both associated with genes located on the N-strand. None of the most frequently used codons (Ile, Leu-TTA, Met, Phe, Asn and Tyr) exhibit high INP% scores, whereas *tRNA-Phe* has the most variable tRNAs in both Pentatomomorpha and Lygaeoidea mtDNA.

### Ribosomal RNAs

**Figure 4:**
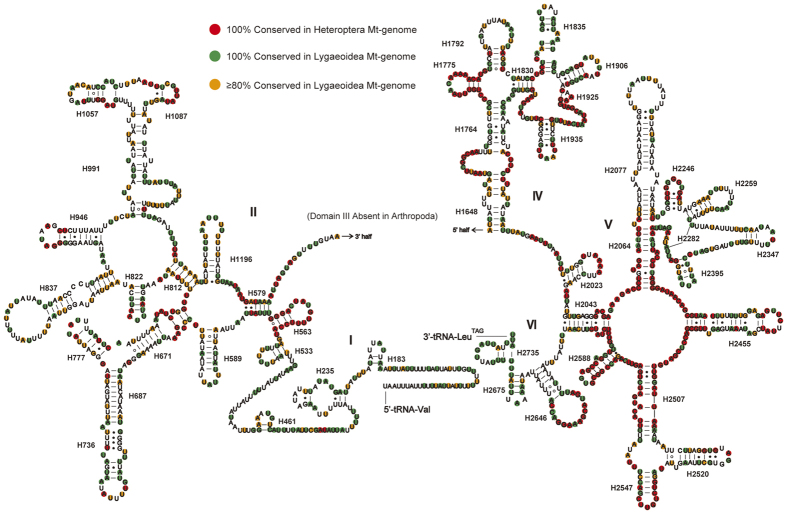
Predicted secondary structure of the mitochondrial 16S rRNA of *Panaorus albomaculatus*. Canonical Watson-Crick interactions are represented by a dash, non-canonical G-U interactions are represented by an asterisk, and all other non-canonical interactions are represented by a hollow circle. Roman numerals denote the conserved domain structure. The 100% conserved sites among sequenced true bugs are plotted with red background. The 100% and > 80% conserved sites among sequenced Lygaeoidea species are plotted with green and orange background, respectively.

**Figure 5:**
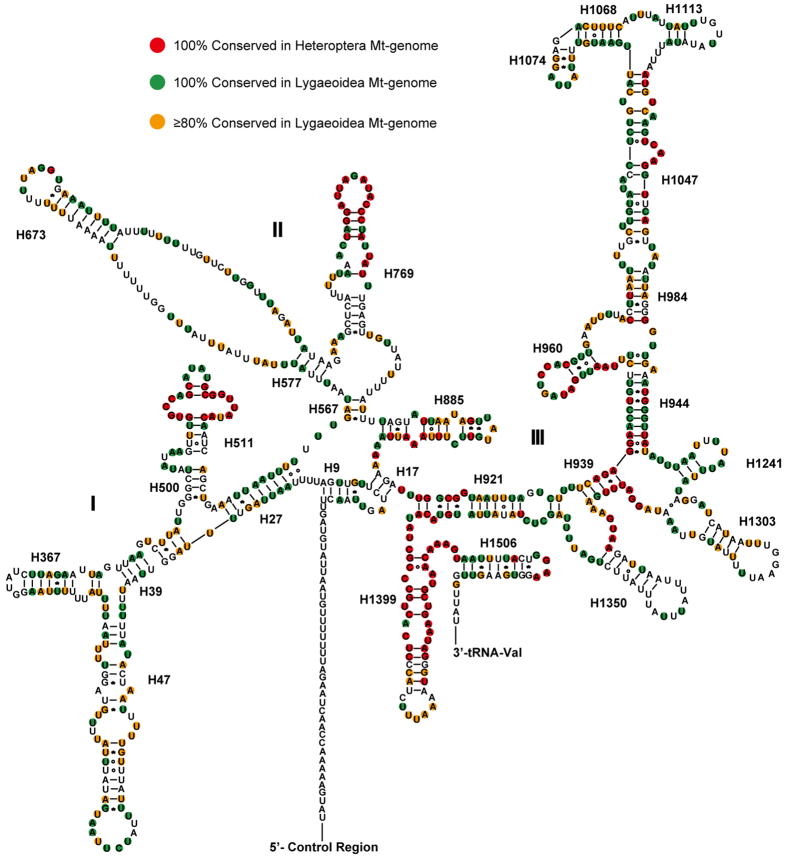
Predicted secondary structure of the mitochondrial 12S rRNA of *Panaorus albomaculatus*. The annotation is the same as for Fig. 4.

We assumed that the rRNA genes in the mt-genome of *P. albomaculatus* start and end at the boundaries of the flanking genes, as is the case in most other insect mt-genomes^22^,^38^. The large subunit (16S) rRNA gene is located between the *tRNA-Val* and *tRNA-Leu (TAG)* genes. The small subunit (12S) rRNA is located between the *tRNA-Val* gene and the control region. The 16S rRNA gene is 1251 bp with an A + T content of 79.9%, and the 12S rRNA gene is 787 bp with an A + T content of 76.8%. The secondary structure of *P. albomaculatus* 16S rRNA contains six structural domains (domain III is absent as in other arthropods) and 45 helices (Fig. 4), whereas the secondary structure of 12S rRNA contains three domains and 27 helices (Fig. 5).

Based on the alignment of sequenced species between Heteroptera and Lygaeoidea, domains IV and V of the large subunit (16S) rRNA are more conserved than other three domains (I, II, and VI). Six helices (H563, H1775, H2064, H2507, H2547, and H2588) are highly conserved in both sequence (with 2–7 nucleotide substitutions) and secondary structure among sequenced heteropterans. In the variable domains II and VI, we found no conserved helices within heteropterans, but helices H579, H822, and the terminal loop of H2646 are highly conserved with 0–3 nucleotide substitutions in Lygaeoidea mtDNA. In domain IV, helices from H1830 to H1906 are highly conserved among Lygaeoidea. Our proposed secondary structure of helix H1835 is based on the model proposed by Buckley *et al*.^39^ and Cannone *et al*.^40^. In domain V, most helices are conserved among insect mtDNAs, except for helices H2077 and H2347, which are highly divergent in both length and secondary structure^39^. Helix H2077 has no apparent conserved motifs, but it contains an 18-paired-base stem and two loops in *P. albomaculatus*. Helix H2347 is composed of a short stem of the terminal 3 paired bases, which is similar to the structure proposed for *Chauliops fallax* (Hemiptera)^10^ and *Zygaena sarpedon lusitanica* (Lepidoptera)^41^.

Domain III of the 12S rRNA is among the most conserved regions among heteropterans in terms of sequence and secondary structure. Domains I and II are much less conserved than domain III, as most helices are highly variable among the studied taxa and are difficult to align. The primary exceptions are the distal sections of H511 and H769, which are highly conserved across insects^42^,^43^. In contrast to H511 and H769, there are considerable differences in the sequence and length of H47 across insects^43^,^44^, and alignments of this region across insects are ambiguous (partly because of its high AT content). We could not detect or predict a consistent secondary structure for H47 across the available insect 12S rRNA sequences. In *P. albomaculatus*, a possible secondary structure of H47 includes a long stem, two internal loops, and 10 bp terminal loop, which is similar to the model proposed by Niehuis *et al*.^42^. The nucleotide sequence between helices H577 and H673 suggests no obvious secondary folding, as in other heteropterans^10^,^27^. In domain III, there are several possible secondary structures for helices H1047, H1068, H1074, and H1113; this complex region likely contains several non-canonical base pairs^43^,^44^. Nonetheless, the sequence and predicted secondary structure of this region are highly conserved within Lygaeoidea. Helix H1399 is the most conserved helix of 12S rRNA among true bugs, except for its terminal loop.

### Non-coding regions

There are only five non-coding regions in the mt-genome of *P. albomaculatus*, and most of them are shorter than 10 bp (Table 1). The largest non-coding region (1,853 bp) is located between the 12S rRNA and the *tRNA-Ile* (I)- *tRNA-Gln* (Q)- *tRNA-Me*t (M) gene cluster (Fig. 1), as frequently found in other insects^15^,^43^. This region is considered to be the control region as it contains both an origin of transcription and replication^9^,^45^.

**Figure 6:**
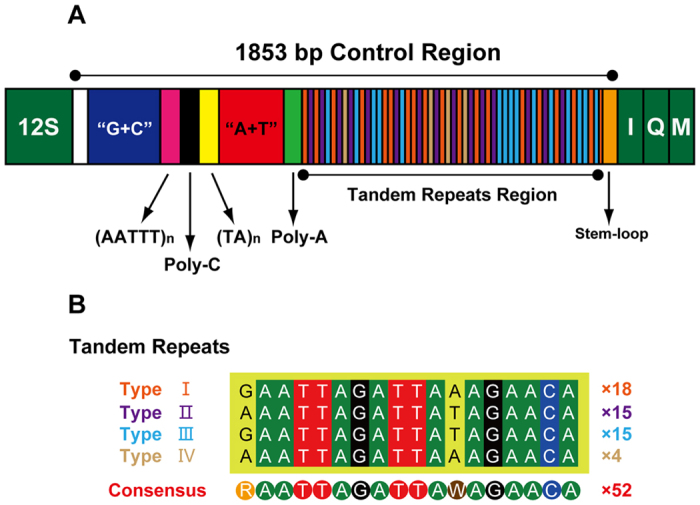
Control region of *Panaorus albomaculatus* mitochondrial genome. (**A**) Structure elements found in the control region of *Panaorus albomaculatus*. The control region flanking genes 12S rRNA, *tRNA-I, tRNA-Q,* and *tRNA-M* are represented in green boxes; “(AATTT)n” (magenta) and “(TA)n” (yellow) indicates the microsatellite-like repeat region; “A + T” (red) indicates high A + T content region; 53 boxes with four different colors indicate the tandem repeats region; the orange-red, purple, blue, and khaki boxes represent four different types of repeat units; orange boxes represent the stem-loop region. (**B**) The alignments of four different types of repeat units were generated by plotting the identities in different colors; numbers indicate the copy number.

The control region is highly variable in length at all taxonomic scales within insects; most of the length variation is a result of variable numbers of tandem repeats^13^,^16^. Tandemly repeated sequences are commonly reported from the control region of most insect orders^46^, although both the size and copy number of tandem repeat units differ greatly across groups^29^. The repeat units of insect mt-genomes range from 8 bp to more than 700 bp, and the copy number of tandem repeats ranges from two to nearly 50^16^,^47^. However, the control region of *P. albomaculatus* has a novel feature not previously observed in other insect mt-genomes. The majority of the control region is made up of a 949 bp tandem-repeat region, which contains 53 tandem duplications including 52 repeat units (of 18 bp each) and a partial copy of the repeat (13 bp) (Fig. 6). This is the largest number of tandem repeats reported in insect mt-genomes to date. In addition, the nucleotide composition of this tandemly repeated region on the J-strand is strongly biased in composition toward A (A = 54.3%, T = 25.5%, C = 5.5%, G = 14.7%), which results in a higher AT-skew value for control region compared to the other genes of *P. albomaculatus* mt-genome (see Supplementary Table S2).

The remainder of the control region can be divided into five sections (Fig. 6A): (1) the 5′-end of the control region contains a 321-bp region with 32.1% G + C content (higher than the average for the entire mt-genome); (2) a 8-bp poly-C structure is located between two microsatellite-like repeat regions ((AATTT)_3_ and (TA)_5_, respectively); (3) a 103-bp region with very high A + T composition (90.3%), as also found in the control region of other insects^48^,^49^; (4) a 8-bp poly-A structure is located 34-bp upstream of the tandem repeat region; and (5) a potential stem-loop secondary structure, with a ‘TATA’ element at the 5′ end (see Supplementary Figure S3).

Our comparative analyses of control regions among sequenced Pentatomomorpha mt-genomes revealed four distinct types of control region organization, which are summarized in Supplementary Figure S4. In some species of Pentatomomorpha, the control region contains four different motifs as summarized for arthropods^50^: tandem repeats, a poly-T stretch, a subregion of even higher A + T content, and stem-loop structures (e.g., *Chauliops fallax* in Supplementary Figure S4A). However, in most mt-genomes of Pentatomomorpha, tandem repeats occupy the majority of the control region, and two different types (see Supplementary Figure S4B) or identical tandem repeat units (Supplementary Figure S4C) are interrupted by a non-coding region^23^,^51^. A few control regions do not contain all four motifs or have only one; for example, *Kleidocerys resedae resedae*^13^ has just a stem-loop structure (see Supplementary Figure S4D). These various elements of the control region can provide valuable phylogenetic information within specific groups^29^,^52^,^53^.

### Tandem repeats

The origin and persistence of tandem repeats is thought to involve slipped-strand mispairing during mtDNA replication^15^,^19^. Other mechanisms for repetitive sequences generation, such as recombination, are considered absent or rare in animal mt-genomes^54^. In *P. albomaculatus*, the repeat units consist of four different patterns with two variable sites (Fig. 6B): Type I (18 copies), Type II (15 copies), Type III (15 copies), and Type IV (4 copies). These four types are indicated by different colors in Fig. 6A.

These four types of repeat units occur throughout the tandem repeats region, with the following patterns (Fig. 6): (1) A partial copy Type I (GAATTAGATTAAA) is located at the 3′-end of the tandem repeats region, so Type I repeats occur at both the start and end of the repeat unit. (2) Most copies of Type II are located immediately downstream of Type I repeats, and all copies of Type IV are followed by Type II repeats. (3) Type III repeats are located between Type II and Type I repeats. (4) There are only four copies of Type IV repeats, but the other three types are all duplicated at least 15 times.

**Figure 7:**
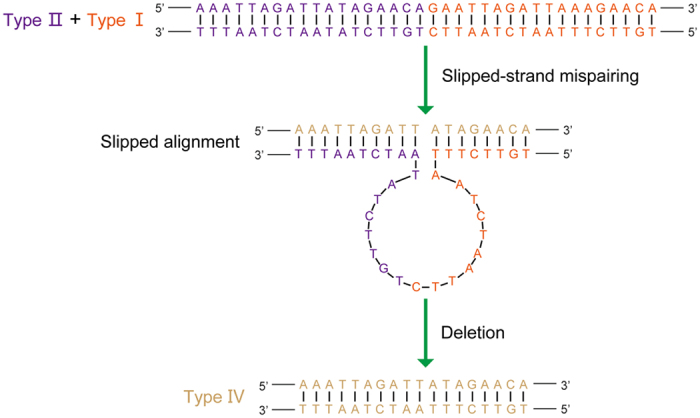
Proposed mechanism involved in the generation of Type IV induced by slipped-strand mispairing. Slipped alignment produces a loop between the repeats of Type II and Type I, and results in a deletion after completion of replication.

Considering these patterns of repeats, we suggest that the ancestral sequence consisted of a Type I-Type II-Type III-Type I cluster. The current distribution can then be generated from this ancestral sequence via replication slippage. Type IV repeats may be a product of slipped-strand mispairing from deletion of the unpaired section between Type II and Type I (Fig. 7). Duplication rates have been shown to be higher than deletion rates in several previous studies^19^,^55^, which likely accounts for the lower number of Type IV repeats compared to the other three types.

There is a stem-loop structure, which is generally thought to be involved in the origin of replication^15^, located 34-bp downstream of the tandem repeats region (see Supplementary Figure S3). The significant number of tandem repeats adjacent to the origin of replication may indicate that they promote their own copy number amplification.

### Phylogenetic analyses

**Figure 8:**
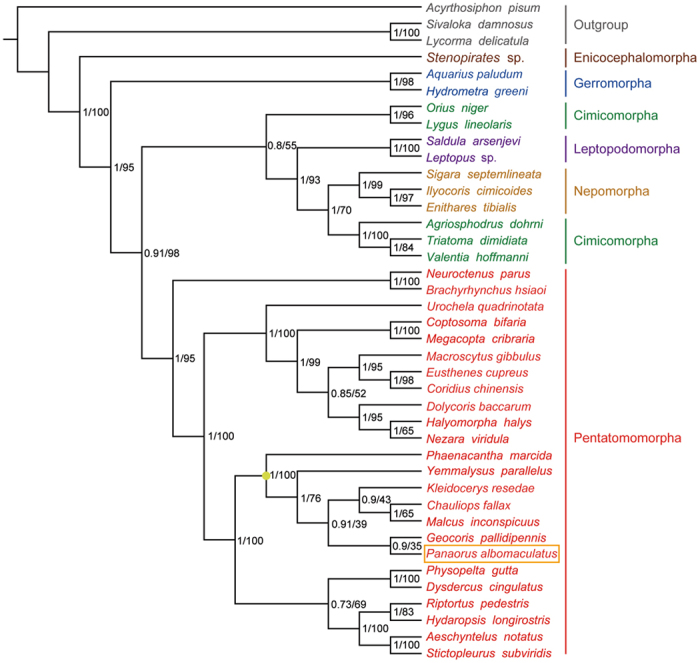
Phylogenetic tree inferred from the sequences of 13 protein coding genes of the mitochondrial genomes of 37 heteropterans and 3 outgroups. Numbers at the nodes are Bayesian posterior probabilities (left) and ML bootstrap values (right). The orange box indicates the position of *Panaorus albomaculatus*. The yellowish dot on the tree indicates the clade of Lygaeoidea.

We conducted phylogenetic analyses on the mt-genomes of 37 heteropteran species and 3 outgroup hemipteran insect species (*Acyrthosiphon pisum*, *Sivaloka damnosa,* and *Lycorma delicatula*). Maximum likelihood (ML) and Bayesian inference (BI) analyses yielded fully resolved trees with identical topologies (Fig. 8). The resulting phylogeny of Heteropteran infraordinal relationships is similar to that of Li *et al*.^56^. We found Enicocephalomorpha to be the sister group to all the remaining heteropterans, with high support values from both BI and ML analyses. In Fig. 8, all infraorders are monophyletic except Cimicomorpha. The non-monophyly of Cimicomorpha is incongruent with previous studies^57^,^58^; a more thorough taxon sampling of taxa within Cimicomorpha is needed to test these competing hypotheses. In Pentatomomorpha, our results support the groupings (Aradoidea + (Pentatomoidea + (Lygaeoidea + (Pyrrhocoroidea + Coreoidea)))), which have been widely supported in previous studies^1^,^10^,^23^. The monophyly of each superfamily within Pentatomomorpha is strongly supported in our analyses. The taxonomic status of Rhyparochromidae within Lygaeoidea is a highly contentious issue^1^,^5^,^6^,^8^. In our analyses, both BI and ML analyses provide strong support that Rhyparochromidae is not the sister group to the remaining Lygaeoidea, which is consistent with previous studies based on molecular data^4^,^5^,^6^. We also recovered the groups (Colobathristidae + (Berytidae + ((Lygaeidae + Malcidae) + (Geocoridae + Rhyparochromidae)))), but without strong support from either BI or ML analyses. We suggest that more thorough sampling of taxa will be needed to adequately resolve the family relationships within Lygaeoidea.

## Conclusions

We present the first description of a complete mitochondrial genome from a species of Rhyparochromidae, a large and diverse group within Heteroptera^3^. Comparative analysis of tRNA gene families showed that the level of conservation of many tRNAs was not consistent between sequenced Pentatomomorpha and Lygaeoidea mt-genomes, and most tRNA genes encoded on the J-strand are more conserved than those encoded on the N-strand. The control region of *P. albomaculatus* has novel patterns and large numbers of tandem repeats, including 53 tandem duplications, which can be explained by a replication slippage model. Phylogenomic analysis based on mt-genomes supported findings from nuclear genes that Rhyparochromidae is not the sister group to all the remaining Lygaeoidea, in contrast to the conclusions from morphological studies. This study should facilitate additional studies on the evolution and phylogeny of Rhyparochromidae, and on the evolution of tandem repeatswithin control regions of mt-genomes.

## Materials and Methods

### Specimen collection

We collected adult specimens of *Panaorus albomaculatus* from Nankai University (39°6.016N, 117°9.977E), Tianjin, China, on July 7th, 2008. We preserved specimens in 95% ethanol and stored them at −20 °C until tissues were used for DNA extraction. Voucher specimens are deposited in the Insect Molecular Systematic Lab, Institute of Entomology, College of Life Sciences, Nankai University, Tianjin, China.

### PCR amplification and sequencing

We extracted genomic DNA from thoracic muscle tissue using the CTAB-based method^59^. We generated the entire mt-genome of *Panaorus albomaculatus* by amplification of four overlapping PCR fragments. We designed primers from the sequenced fragments, modifying primers from previous studies^13^ (see Supplementary Table S4). We performed PCR reactions using TaKaRa LA *Taq* under the following conditions: 1 min initial denaturation at 94 °C, followed by 30 cycles of 20 s at 94 °C, 1 min at 50 °C, and 2–8 min at 68 °C, and a final elongation for 10 min at 72 °C. We separated PCR products using electrophoresis in 1% agarose gels, purified the fragments, and then sequenced both strands of each fragments using a ABI 3730XL capillary sequencer with the BigDye Terminator Sequencing Kit (Applied Bio Systems).

### Sequence analysis and annotation

We proofread the raw sequence files and then aligned them into contigs using BioEdit version 7.0.5.2^60^. We identified PCGs using ORF Finder as implemented at the NCBI website (http://www.ncbi.nlm.nih.gov/gorf/gorf.html), using invertebrate mitochondrial genetic codes. We compared the boundaries of our predicted PCGs and rRNAs with published insect mt-genomes, and the alignments were produced with CLUSTAL X version 1.83^61^. We identified tRNA genes using tRNAscan-SE version 1.21^62^, with the invertebrate mitochondrial codon predictors, and a cove cut-off score of 5. A few of the tRNA genes that could not be detected using tRNAscan-SE were determined through comparing the sequences to other heteropterans. Our analyses of nucleotide composition and codon usage were conducted using MEGA 6.0^63^. We calculated nucleotide skew statistics using the following formulas: AT skew = [A−T]/[A + T] and GC skew = [G−C]/[G + C]^33^. Tandem repeats were identified using the Tandem Repeats Finder server (http://tandem.bu.edu/trf/trf.html)^64^, and the stem-loop structure was inferred using the mfold web server (http://mfold.rna.albany.edu/)^65^.

### Prediction of secondary structure of rRNAs

Our secondary structural predictions for 12S rRNA and 16S rRNA are based on previously published models of secondary structure in other insects, including *Chauliops fallax* (Hemiptera: Malcidae)^10^, *Drosophila melanogaster* (Diptera: Drosophilidae)^40^, *Apis mellifera* (Hymenoptera: Apidae)^44^, *Manduca sexta* (Lepidoptera: Sphingidae)^43^, and *Stenopirates* sp. (Hemiptera: Enicocephalidae)^56^. We followed Gillespie *et al*.^44^ and Cameron *et al*.^43^ in naming stem-loop structures. We inferred homology within regions of low sequence similarity using the mfold web server^65^.

### Phylogenetic analyses

We conducted phylogenetic analyses on the 40 complete or nearly complete mt-genomes of true bugs that have sequences in GenBank, as well as three species from Sternorrhyncha and Auchenorrhyncha which we used as our outgroup (see Supplementary Table S5). We aligned PCGs based on amino acid sequence alignment using MUSCLE in MEGA version 6.0^63^. Our alignments of individual genes excluded the stop codon and the third codon. We used GPU MrBayes^66^ for Bayesian inference and raxmlGUI 1.5^67^ for ML phylogenetic analysis. All analyses were based on the GTR + I + Γ model of sequence evolution, as selected using on Modeltest version 3.7^68^. We conducted two simultaneous runs of 10,000,000 generations each for the Bayesian analyses, with a burn-in of 2,500,000 generations and sampling every 100 generations. In ML analyses, we conducted 1000 bootstrap replicates with thorough ML searches.

## Additional Information

**How to cite this article**: Li, T. *et al*. A Mitochondrial Genome of Rhyparochromidae (Hemiptera: Heteroptera) and a Comparative Analysis of Related Mitochondrial Genomes. *Sci. Rep.* **6**, 35175; doi: 10.1038/srep35175 (2016).

## Supplementary Material

Supplementary Information

## Figures and Tables

**Table 1:** Organization of the *Panaorus albomaculatus* mitochondrial genome.

Gene	Strand	Position	Anticodon	Size (bp)	Start codon	Stop codon	Intergenic Nucleotides^a^
tRNA-Ile	J	1–65	GAT	65			
tRNA-Gln	N	63–131	TTG	69			−3
tRNA-Met	J	131–198	CAT	68			−1
ND2	J	199–1170		972	ATT	TAA	0
tRNA-Trp	J	1248–1310	TCA	63			77
tRNA-Cys	N	1311–1372	GCA	62			0
tRNA-Tyr	N	1373–1434	GTA	62			0
CO1	J	1436–2974		1539	TTG	TAA	1
tRNA-Leu	J	2970–3034	TAA	65			−5
CO2	J	3035–3701		667	ATT	T-	0
tRNA-Lys	J	3702–3772	CTT	71			0
tRNA-Asp	J	3772–3834	GTC	63			−1
ATPase8	J	3835–3990		156	ATT	TAA	0
ATPase6	J	3984–4658		675	ATG	TAA	−7
CO3	J	4645–5431		787	ATG	T-	−14
tRNA-Gly	J	5432–5498	TCC	67			0
ND3	J	5499–5849		351	ATA	TAG	0
tRNA-Ala	J	5848–5910	TGC	63			−2
tRNA-Arg	J	5910–5971	TCG	62			−1
tRNA-Asn	J	5972−6042	GTT	71			0
tRNA-Ser	J	6042−6110	GCT	69			−1
tRNA-Glu	J	6110–6174	TTC	65			−1
tRNA-Phe	N	6174–6237	GAA	64			−1
ND5	N	6237–7943		1707	ATT	TAA	−1
tRNA-His	N	7944–8005	GTG	62			0
ND4	N	8006–9317		1312	ATG	T-	0
ND4L	N	9311–9586		276	GTG	TAA	−7
tRNA-Thr	J	9589–9651	TGT	63			2
tRNA-Pro	N	9652–9714	TGG	63			0
ND6	J	9717–10190		474	ATC	TAA	2
Cytb	J	10183–11316		1134	ATG	TAG	−8
tRNA-Ser	J	11315–11384	TGA	70			−2
ND1	N	11365–12321		957	ATT	TAA	−20
tRNA-Leu	N	12322–12386	TAG	65			0
16S rRNA	N	12387–13637		1251			0
tRNA-Val	N	13638–13705	TAC	68			0
12S rRNA	N	13706–14492		787			0
Control		14493–16345		1853			0

^a^Numbers correspond to nucleotides separating a gene from an upstream one; negative numbers indicate that adjacent genes overlap.
