# Supplementary material for: A Mitochondrial Genome of Rhyparochromidae (Hemiptera: Heteroptera) and a Comparative Analysis of Related Mitochondrial Genomes
Source: Sci Rep. 2016 Oct 19;6:35175. doi: 10.1038/srep35175 (PMC5069475; doi:10.1038/srep35175)

# A Mitochondrial Genome of Rhyparochromidae (Hemiptera: Heteroptera) and a Comparative Analysis of Related Mitochondrial Genomes

Teng Li^1, 2^, Jie Yang^2^, Yinwan Li^2^, Ying Cui^2^, Qiang Xie^2^, Wenjun Bu^2^*, and David M. Hillis^3^*

^1^Institute of Zoology and Developmental Biology, College of Life Sciences, Lanzhou University, 222 Tianshui South Road, Lanzhou 730000, China

^2^Institute of Entomology, College of Life Sciences, Nankai University, 94 Weijin Road, Tianjin 300071, China

^3^Department of Integrative Biology, University of Texas at Austin, Austin, TX 78712, USA

*Corresponding author

Email addresses:

TL: teng.chn@gmail.com

JY: yangjiejnxy0601@163.com

YL: [youfulei@126.com](mailto:youfulei@126.com)

YC: cql8179270@126.com

QX: qiangxie@nankai.edu.cn

WB: [wenjunbu@nankai.edu.cn](mailto:wenjunbu@nankai.edu.cn)

DMH: dhillis@austin.utexas.edu

Tel: 0086-22-23498957, Fax: 0086-22-23498957.

## Supplementary Table S1. Start and stop codons of 13 protein-coding genes of 24 Pentatomomorpha species.

| Species | ATP6 | | ATP8 | | COI | | COII | | COIII | | CytB | | ND1 | | | ND2 | | ND3 | | | ND4 | | ND4L | | ND5 | | ND6 | | |
| --- | --- | --- | --- | --- | --- | --- | --- | --- | --- | --- | --- | --- | --- | --- | --- | --- | --- | --- | --- | --- | --- | --- | --- | --- | --- | --- | --- | --- | --- |
|  | Start | Stop | Start | Stop | Start | Stop | Start | Stop | Start | Stop | Start | Stop | Start | Stop | Start | | Stop | | Start | Stop | Start | Stop | Start | Stop | Start | Stop | | Start | Stop |
| *Brachyrhynchus hsiaoi* | ATG | TAA | ATA | TAA | TTG | TAG | TTG | T- | ATG | T- | ATG | TA- | TTG | TAA | ATA | | T- | | ATA | TA- | ATG | TAA | ATT | TAA | ATG | T- | | ATT | TAA |
| *Neuroctenus parus* | ATG | TAA | ATA | TAA | TTG | TAA | ATA | T- | ATG | TAA | ATG | T- | ATT | TAA | ATA | | T- | | ATA | TA- | ATG | T- | ATT | TAA | ATT | T- | | ATA | TAA |
| *Coptosoma bifaria* | ATG | TAA | ATA | TAA | TTG | TAA | ATA | T- | ATG | TAA | ATG | TAG | ATG | TAA | ATT | | TAA | | ATA | TAG | ATG | TAA | TTG | TAA | ATT | TAA | | ATG | TAA |
| *Coridius chinensis* | ATG | TAA | TTG | TAA | TTG | TAA | ATA | T- | ATG | TAA | ATG | TAA | GTG | TAA | ATC | | TAA | | ATA | TAA | ATG | TAG | ATT | TAA | ATT | TAA | | GTG | TAA |
| *Dolycoris baccarum* | ATG | TAA | ATA | TAA | TTG | T- | ATA | T- | ATG | TAA | ATG | TAA | TTG | TAA | ATT | | TAA | | ATA | TAA | ATG | TAA | ATT | TAA | ATA | TAA | | ATA | TAA |
| *Eusthenes cupreus* | ATG | TAG | ATG | TAA | TTG | TA- | ATA | T- | ATG | T- | ATG | TAG | GTG | TAA | ATA | | TAA | | ATT | TAG | ATG | TAA | ATT | TAA | ATA | TAA | | TTG | TAA |
| *Halyomorpha halys* | ATG | TAA | ATA | TAA | TTG | TAA | ATA | T- | ATG | TAA | ATG | TAA | TTG | TAA | ATT | | TAA | | ATA | TAA | ATG | TAA | ATT | TAA | ATT | TAA | | ATG | TAA |
| *Macroscytus gibbulus* | ATG | TAG | ATA | TAA | TTG | TA- | ATA | T- | ATG | T- | ATG | TAG | GTG | TAA | ATCA | | TAA | | ATT | TAG | ATG | TAG | ATT | TAA | ATA | T- | | ATA | TAA |
| *Megacopta cribraria* | ATG | T- | ATA | TAA | TTG | TAA | ATA | T- | ATG | T- | ATG | TAA | GTG | TAA | ATT | | TAA | | ATA | TAA | ATG | T- | ATT | TAA | TTG | T- | | TTG | T- |
| *Nezara viridula* | ATG | TAA | TTG | TAA | TTG | TAA | ATA | T- | ATG | TAA | ATG | TAA | CTG | TAA | ATT | | TAA | | ATT | T- | ATG | TAG | ATT | TAA | ATT | T- | | ATA | TAA |
| *Urochela quadrinotata* | ATG | TAA | ATG | TAA | TTG | TAA | ATC | T- | ATG | T- | ATG | TAA | ATT | TAA | ATT | | TAA | | ATC | TAG | ATG | TAG | ATT | TAA | ATT | TAA | | ATG | TAA |
| ***Chauliops fallax*** | ATG | TAA | ATA | TAA | TTG | TAA | ATT | T- | ATG | T- | ATG | TAG | ATT | TAA | ATA | | TAA | | ATA | TAA | ATG | TAA | ATT | TAA | ATT | TAA | | ATC | TAA |
| ***Geocoris pallidipennis*** | ATG | TAA | ATC | TAA | TTG | TAA | GTG | T- | ATG | T- | ATG | TAG | ATA | TAG | ATC | | TAA | | ATT | TAA | ATG | TAA | GTG | TAA | ATT | TAA | | ATT | TAA |
| ***Kleidocerys resedae*** | ATG | TAA | ATA | TAA | TTG | TAA | ATA | T- | ATG | T- | ATG | TAG | ATC | TAA | ATA | | TAA | | ATA | TAG | ATG | TAA | ATT | TAA | ATA | T- | | ATT | TAA |
| ***Malcus inconspicuus*** | ATG | TAA | ATT | TAA | TTG | TAA | ATA | T- | ATG | T- | ATG | TAG | ATT | TAA | ATC | | TAA | | ATT | TAG | ATG | TAA | ATT | TAA | ATT | TAA | | ATA | TAA |
| ***Panaorus albomaculatus*** | ATG | TAA | ATT | TAA | TTG | TAA | ATT | T- | ATG | T- | ATG | TAG | ATT | TAA | ATT | | TAA | | ATA | TAG | ATG | T- | GTG | TAA | ATT | TAA | | ATC | TAA |
| ***Phaenacantha marcida*** | ATG | TAA | ATT | TAA | TTG | TAA | ATA | T- | ATG | TAA | ATG | TAG | ATA | TAA | ATT | | TAA | | ATC | TAG | ATG | TAG | TTG | TAA | ATT | TAA | | ATT | TAA |
| ***Yemmalysus parallelus*** | ATT | TAA | ATT | TAA | TTG | T- | ATG | T- | ATG | T- | ATG | TAG | ATT | TAA | ATA | | TAA | | ATA | TAA | ATG | TAA | ATT | TAA | ATA | TAA | | ATA | TAA |
| *Dysdercus cingulatus* | ATG | TAA | ATT | TAA | TTG | TAA | ATG | T- | ATG | T- | ATG | TAG | ATA | TAA | ATA | | TAA | | ATT | TAA | ATG | TAA | ATT | TAA | ATA | T- | | ATA | TAA |
| *Physopelta gutta* | ATG | TAA | ATA | TAA | TTG | TAA | ATG | T- | ATG | T- | ATG | TAA | ATT | TAA | ATT | | TAA | | ATA | TAG | ATG | TAA | ATT | TAA | ATT | TAA | | ATT | TAA |
| *Aeschyntelus notatus* | ATG | TAA | ATT | TAA | TTG | TAA | ATT | T- | ATG | T- | ATG | TAG | ATA | TAA | ATG | | TAA | | ATT | T- | ATG | TAA | ATT | TAA | ATT | TAA | | ATA | TAA |
| *Hydaropsis longirostris* | ATG | TAA | ATT | TAA | TTG | TAA | ATT | T- | ATG | T- | ATA | TAG | ATT | TAA | ATA | | TAA | | ATA | TAG | ATG | T- | ATT | TAA | ATG | TAA | | ATT | TAA |
| *Riptortus pedestris* | ATG | TAA | ATC | TAA | TTG | TAA | ATC | T- | ATG | T- | ATA | TAA | ATA | TAA | ATG | | T- | | ATA | T- | ATG | TAG | ATT | TAA | ATG | TAA | | ATA | TAA |
| *Stictopleurus subviridis* | ATG | TAA | ATT | TAA | TTG | TAG | ATT | T- | ATG | T- | ATG | TAG | ATA | TAG | ATC | | TAA | | ATT | TAA | ATG | TAG | ATT | TAA | ATT | TAA | | ATA | TAA |

Note: Seven species of Lygaeoidea are indicated with bold characters.

## Supplementary Table S2. Nucleotide composition of the *Panaorus albomaculatus* mitochondrial genome.

| Feature | Length (bp) | A% | C% | G% | T% | A+T% | AT-Skew | GC-Skew |
| --- | --- | --- | --- | --- | --- | --- | --- | --- |
| Whole genome | 16345 | 44.4 | 14.5 | 9.5 | 31.6 | 76.0 | 0.17 | -0.21 |
| Protein-coding genes | 11007 | 33.3 | 12.7 | 12.2 | 41.8 | 75.1 | -0.11 | -0.02 |
| Protein-coding genes-J | 6755 | 38.0 | 15.9 | 10.7 | 35.4 | 73.4 | 0.04 | -0.20 |
| Protein-coding genes-N | 4252 | 25.8 | 7.7 | 14.6 | 51.9 | 77.7 | -0.34 | 0.31 |
| First codon position | 3658 | 36.9 | 11.7 | 17.0 | 34.4 | 71.3 | 0.04 | 0.18 |
| Second codon position | 3658 | 21.2 | 17.4 | 14.4 | 47.0 | 68.2 | -0.38 | -0.09 |
| Third codon position | 3658 | 41.6 | 9.1 | 5.4 | 43.9 | 85.5 | -0.03 | -0.26 |
| tRNA genes | 1440 | 39.2 | 9.4 | 12.6 | 38.8 | 78.0 | 0.01 | 0.15 |
| tRNA genes-J | 925 | 41.1 | 10.8 | 10.9 | 37.2 | 78.3 | 0.05 | 0.00 |
| tRNA genes-N | 515 | 35.9 | 6.8 | 15.7 | 41.6 | 77.5 | -0.07 | 0.40 |
| rRNA genes | 2038 | 29.3 | 7.6 | 13.7 | 49.4 | 78.7 | -0.26 | 0.29 |
| Control region | 1853 | 47.9 | 11.0 | 11.6 | 29.5 | 77.4 | 0.24 | 0.03 |
| ATP6 | 675 | 38.2 | 15.1 | 9.3 | 37.4 | 75.6 | 0.01 | -0.24 |
| ATP8 | 156 | 47.4 | 9.6 | 6.4 | 36.6 | 84.0 | 0.13 | -0.20 |
| COI | 1539 | 34.2 | 17.1 | 14.1 | 34.6 | 68.8 | -0.01 | -0.10 |
| COII | 667 | 38.1 | 19.2 | 10.5 | 32.2 | 70.3 | 0.08 | -0.29 |
| COIII | 787 | 36.5 | 16.4 | 12.8 | 34.3 | 70.8 | 0.03 | -0.12 |
| CytB | 1134 | 37.6 | 17.4 | 10.8 | 34.2 | 71.8 | 0.05 | -0.23 |
| ND1 | 957 | 22.4 | 8.0 | 16.1 | 53.5 | 75.9 | -0.41 | 0.34 |
| ND2 | 972 | 40.8 | 12.9 | 7.9 | 38.4 | 79.2 | 0.03 | -0.24 |
| ND3 | 351 | 37.0 | 15.9 | 10.3 | 36.8 | 73.8 | 0.00 | -0.21 |
| ND4 | 1312 | 25.5 | 7.8 | 14.9 | 51.8 | 77.3 | -0.34 | 0.31 |
| ND4L | 276 | 21.8 | 5.4 | 15.2 | 57.6 | 79.4 | -0.45 | 0.48 |
| ND5 | 1707 | 28.7 | 7.7 | 13.4 | 50.2 | 78.9 | -0.27 | 0.27 |
| ND6 | 474 | 45.6 | 11.6 | 6.1 | 36.7 | 82.3 | 0.11 | -0.31 |
| 12S rRNA | 787 | 28.6 | 8.1 | 15.1 | 48.2 | 76.8 | -0.26 | 0.30 |
| 16S rRNA | 1251 | 29.7 | 7.2 | 12.9 | 50.2 | 79.9 | -0.26 | 0.28 |

## Supplementary Table S3. Relative synonymous codon usage of each amino acid in the *Panaorus albomaculatus* mitochondrial genome.

| **Amino acid** | **Codon** | **N** | **RSCU** | **%** | **Amino acid** | **Codon** | **N** | **RSCU** | **%** |
| --- | --- | --- | --- | --- | --- | --- | --- | --- | --- |
| Phe | UUU | 271 | 1.65 | 7.41 | Tyr | UAU | 164 | 1.72 | 4.48 |
|  | UUC | 58 | 0.35 | 1.59 |  | UAC | 27 | 0.28 | 0.74 |
| Leu | UUA | 337 | 4.04 | 9.21 | His | CAU | 44 | 1.29 | 1.20 |
|  | UUG | 39 | 0.47 | 1.07 |  | CAC | 24 | 0.71 | 0.66 |
| Leu | CUU | 45 | 0.54 | 1.23 | Gln | CAA | 44 | 1.66 | 1.20 |
|  | CUC | 11 | 0.13 | 0.30 |  | CAG | 9 | 0.34 | 0.25 |
|  | CUA | 59 | 0.71 | 1.61 | Asn | AAU | 180 | 1.71 | 4.92 |
|  | CUG | 10 | 0.12 | 0.27 |  | AAC | 30 | 0.29 | 0.82 |
| Ile | AUU | 349 | 1.7 | 9.54 | Lys | AAA | 93 | 1.65 | 2.54 |
|  | AUC | 62 | 0.3 | 1.69 |  | AAG | 20 | 0.35 | 0.55 |
| Met | AUA | 288 | 1.83 | 7.87 | Asp | GAU | 53 | 1.71 | 1.45 |
|  | AUG | 27 | 0.17 | 0.74 |  | GAC | 9 | 0.29 | 0.25 |
| Val | GUU | 84 | 2.05 | 2.30 | Glu | GAA | 71 | 1.8 | 1.94 |
|  | GUC | 7 | 0.17 | 0.19 |  | GAG | 8 | 0.2 | 0.22 |
|  | GUA | 67 | 1.63 | 1.83 | Cys | UGU | 38 | 1.69 | 1.04 |
|  | GUG | 6 | 0.15 | 0.16 |  | UGC | 7 | 0.31 | 0.19 |
| Ser | UCU | 100 | 2.3 | 2.73 | Trp | UGA | 79 | 1.6 | 2.16 |
|  | UCC | 22 | 0.51 | 0.60 |  | UGG | 20 | 0.4 | 0.55 |
|  | UCA | 94 | 2.16 | 2.57 | Arg | CGU | 16 | 1.23 | 0.44 |
|  | UCG | 3 | 0.07 | 0.08 |  | CGC | 1 | 0.08 | 0.03 |
| Pro | CCU | 48 | 1.48 | 1.31 |  | CGA | 32 | 2.46 | 0.87 |
|  | CCC | 28 | 0.86 | 0.77 |  | CGG | 3 | 0.23 | 0.08 |
|  | CCA | 51 | 1.57 | 1.39 | Ser | AGU | 35 | 0.8 | 0.96 |
|  | CCG | 3 | 0.09 | 0.08 |  | AGC | 4 | 0.09 | 0.11 |
| Thr | ACU | 67 | 1.56 | 1.83 |  | AGA | 82 | 1.89 | 2.24 |
|  | ACC | 25 | 0.58 | 0.68 |  | AGG | 8 | 0.18 | 0.22 |
|  | ACA | 79 | 1.84 | 2.16 | Gly | GGU | 65 | 1.29 | 1.78 |
|  | ACG | 1 | 0.02 | 0.03 |  | GGC | 3 | 0.06 | 0.08 |
| Ala | GCU | 48 | 1.67 | 1.31 |  | GGA | 96 | 1.91 | 2.62 |
|  | GCC | 15 | 0.52 | 0.41 |  | GGG | 37 | 0.74 | 1.01 |
|  | GCA | 49 | 1.7 | 1.34 | **Sum** | | **3658** | **62** | **100** |
|  | GCG | 3 | 0.1 | 0.08 |

Note: RSCU, Relative synonymous codon usage; N= frequency of each codon; % = N/3658.

## Supplementary Table S4. Primers designed for *Panaorus albomaculatus* in this study.

| **Primer** | **Sequence (5’-3’)** | **Region** |
| --- | --- | --- |
| COIF | TACAATTTACCGCCTTTAATTCAGCC | F1 |
| COIR | TATTGATAAGACATAGTGGAAGTGG |
|  |  |  |
| CytBF | AAAACAATGATCTTGTAAATCAT | F2 |
| CytBR | AACAGGGGTAACTAAAGGATTGGCT |
|  |  |  |
| BB1F | ATTTGTCGAGATGTTAATAATGGATG | F3 |
| BB1R | TTCAAATAATGGGATGCGTTCTGGTGAT |
|  |  |  |
| BB2F | CATTCAGATGATTAGCAACATTACACGG | F4 |
| BB2R | TACGTTATCAACTGAAAATCCTCCCCAT |

## Supplementary Table S5. Summary of sample information used in present study.

| **Suborder** | **Infraorder/**  **superfamily** | **Family** | **Species** | **Accession Number** |
| --- | --- | --- | --- | --- |
| **Sternorrhyncha** | Aphidoidea | Aphididae | *Acyrthosiphon pisum* | NC_011594 |
| **Auchenorrhyncha** | Fulgoroidea | Fulgoridae | *Lycorma delicatula* | NC_012835 |
|  |  | Issidae | *Sivaloka damnosus* | NC_014286 |
| **Heteroptera** | **Enicocephalomorpha** | Enicocephalidae | *Stenopirates* sp. | NC_016017 |
|  | **Gerromorpha** |  |  |  |
|  | Hydrometroidea | Hydrometridae | *Hydrometra greeni* | NC_012842 |
|  | Gerroidea | Gerridae | *Aquarius paludum* | NC_012841 |
|  | **Nepomorpha** |  |  |  |
|  | Corixoidea | Corixidae | *Sigara septemlineata* | FJ456941 |
|  | Notonectoidea | Notonectidae | *Enithares tibialis* | NC_012819 |
|  | Naucoroidea | Naucoridae | *Ilyocoris cimicoides* | NC_012845 |
|  | **Leptopodomorpha** |  |  |  |
|  | Saldoidea | Saldidae | *Saldula arsenjevi* | NC_012463 |
|  | Leptopodoidea | Leptopodidae | *Leptopus* sp. | FJ456946 |
|  | **Cimicomorpha** |  |  |  |
|  | Cimicoidea | Anthocoridae | *Orius niger* | NC_012429 |
|  | Reduvioidea | Reduviidae | *Triatoma dimidiata* | NC_002609 |
|  |  |  | *Valentia hoffmanni* | NC_012823 |
|  |  |  | *Agriosphodrus dohrni* | NC_015842 |
|  | Miroidea | Miridae | *Lygus lineolaris* | EU401991 |
|  | **Pentatomomorpha** |  |  |  |
|  | Aradoidea | Aradidae | *Neuroctenus parus* | NC_012459 |
|  |  |  | *Brachyrhynchus hsiaoi* | NC_022670 |
|  | Pentatomoidea | Pentatomidae | *Nezara viridula* | NC_011755 |
|  |  |  | *Halyomorpha halys* | NC_013272 |
|  |  |  | *Dolycoris baccarum* | NC_020373 |
|  |  | Tessaratomidae | *Eusthenes cupreus* | NC_022449 |
|  |  | Urostylididae | *Urochela quadrinotata* | NC_020144 |
|  |  | Cydnidae | *Macroscytus gibbulus* | NC_012457 |
|  |  | Dinidoridae | *Coridius chinensis* | JQ739179 |
|  |  | Plataspidae | *Coptosoma bifaria* | NC_012449 |
|  |  |  | *Megacopta cribraria* | NC_015342 |
|  | Lygaeoidea | Berytidae | *Yemmalysus parallelus* | NC_012464 |
|  |  | Colobathristidae | *Phaenacantha marcida* | NC_012460 |
|  |  | Lygaeidae | *Kleidocerys resedae* | KJ584365 |
|  |  | Malcidae | *Malcus inconspicuus* | NC_012458 |
|  |  |  | *Chauliops fallax* | JX839706 |
|  |  | Rhyparochromidae | *Panaorus albomaculatus* | KX216853 |
|  |  | Geocoridae | *Geocoris pallidipennis* | NC_012424 |
|  | Pyrrhocoroidea | Largidae | *Physopelta gutta* | NC_012432 |
|  |  | Pyrrhocoridae | *Dysdercus cingulatus* | NC_012421 |
|  | Coreoidea | Alydidae | *Riptortus pedestris* | NC_012462 |
|  |  | Coreidae | *Hydaropsis longirostris* | NC_012456 |
|  |  | Rhopalidae | *Aeschyntelus notatus* | NC_012446 |
|  |  |  | *Stictopleurus subviridis* | NC_012888 |

## Supplementary Figure S1. Evolutionary rates of 13 protein-coding genes in Pentatomomorpha (A) and Lygaeoidea (B) mitochondrial genomes. The rate of nonsynonymous substitutions (Ka), the rate of synonymous substitutions (Ks), and the ratio of Ka/Ks were calculated for each PCG.


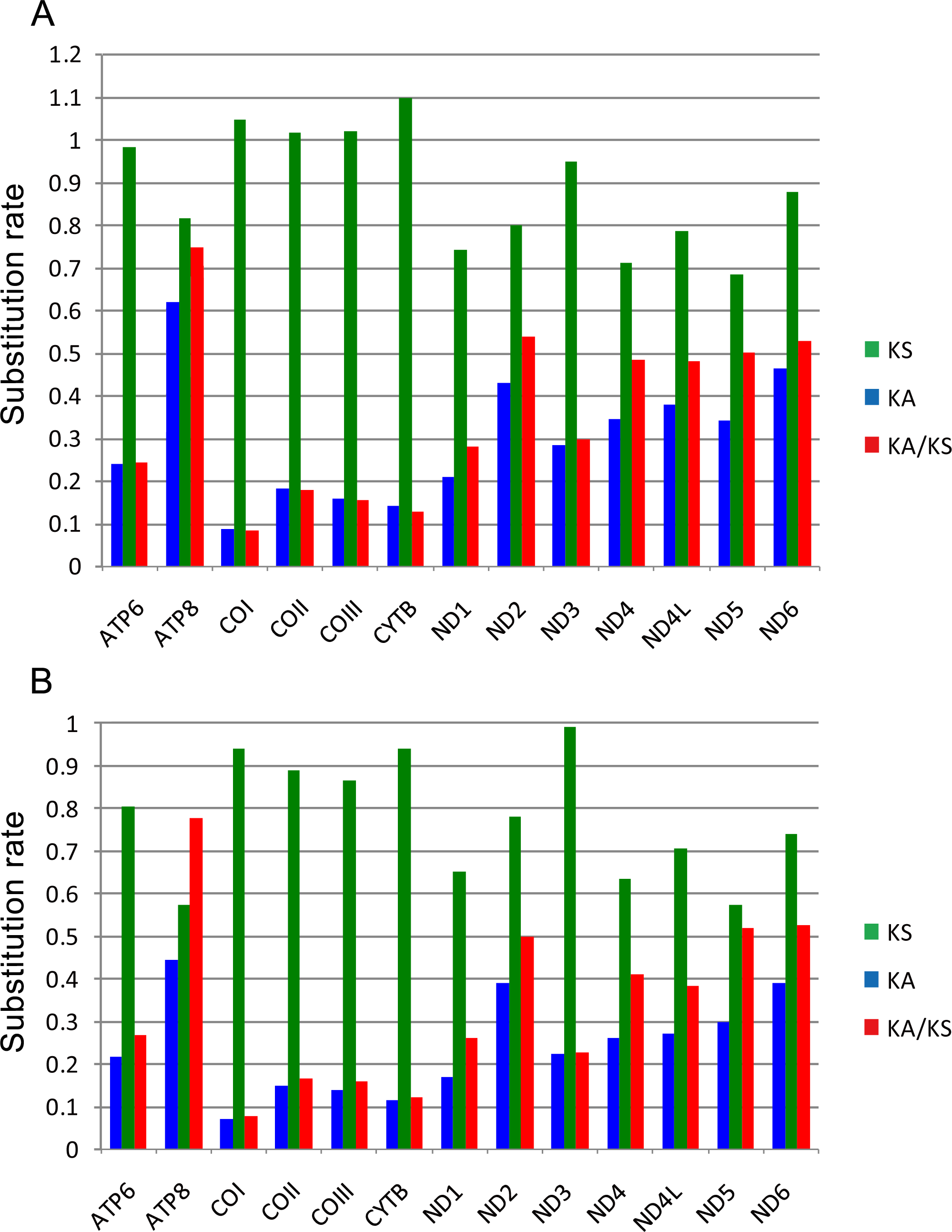


## Supplementary Figure S2. The correlation between the Ka/Ks and the GC content of each protein-coding genes in Pentatomomorpha (A) and Lygaeoidea (B) mitochondrial genomes.


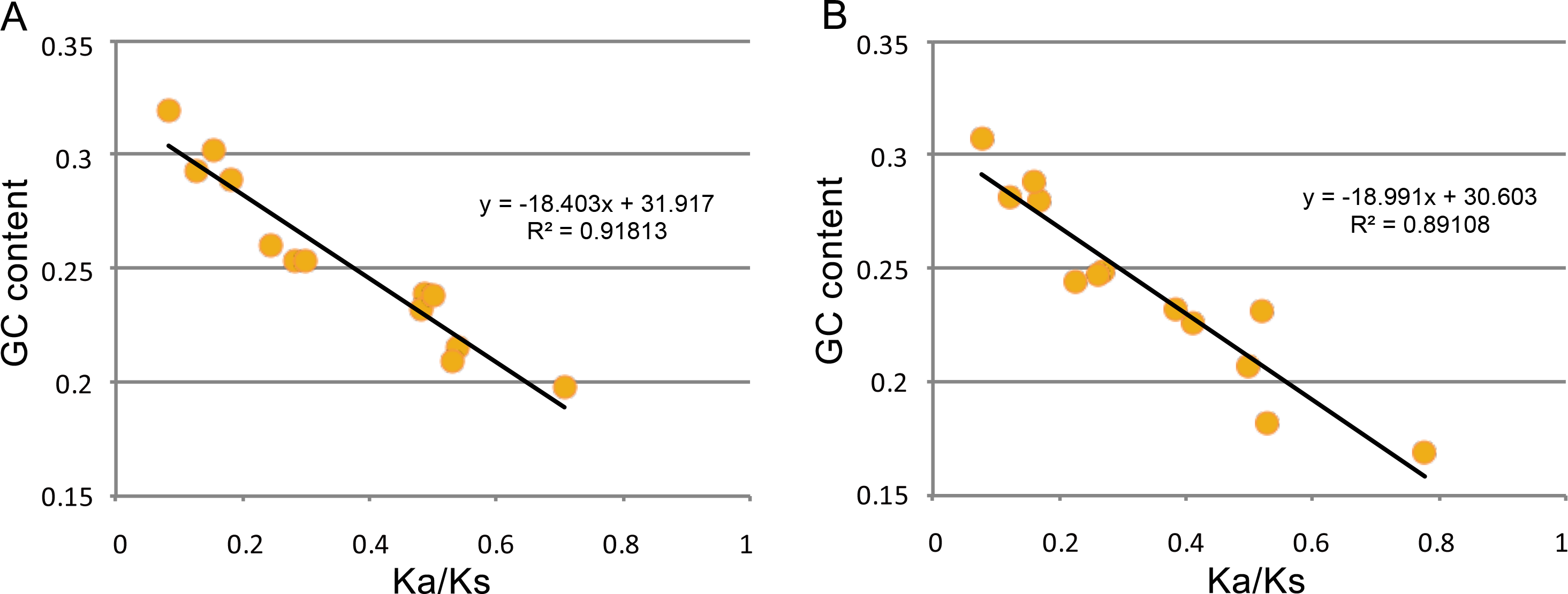


## Supplementary Figure S3. The putative stem-loops structure found in the control region of *Panaorus albomaculatus*. The brown box indicates the highly conserved flanking sequence.


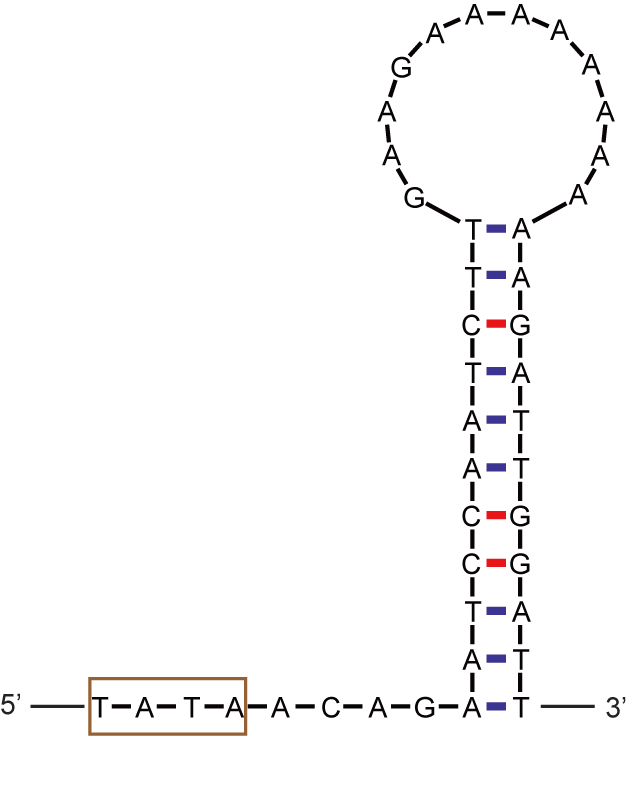


## Supplementary Figure S4. Control regions of some representative species of Pentatomomorpha. (A) The control region of *Chauliops fallax* includes four motifs; (B) The control region of *Halyomorpha halys* includes two different types of tandem repeat sequences; (C) The control region of *Aneurus similis* includes two identical tandem repeats; (D) The control region of *Kleidocerys resedae resedae* has only one motif, a stem-loop structure.


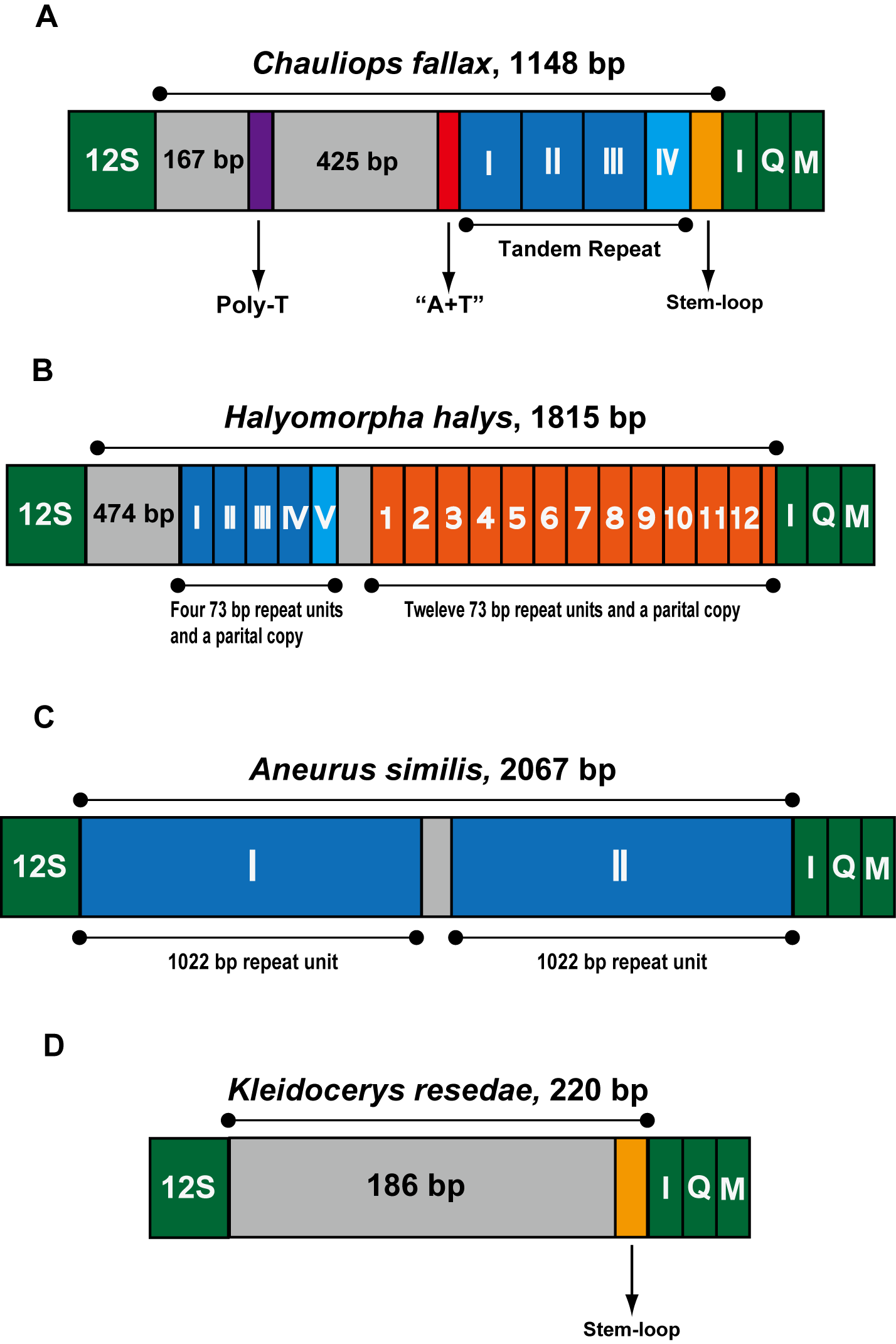

Supplement: Supplementary Information [file srep35175-s1.doc]
